# Supplementary material for: Children can control the expression of masculinity and femininity through the voice
Source: R Soc Open Sci. 2019 Jul 17;6(7):190656. doi: 10.1098/rsos.190656 (PMC6689575; doi:10.1098/rsos.190656)

Supplementary Methods

Stereotypically feminine boy and girl characters

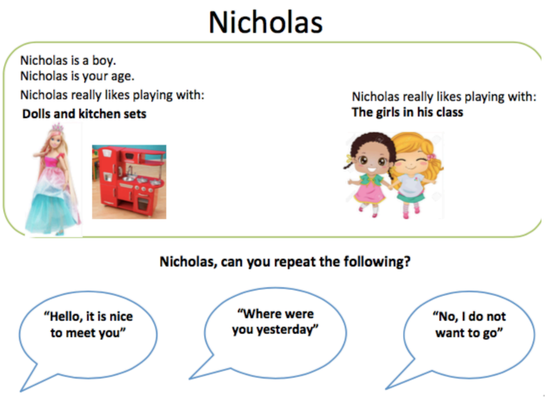

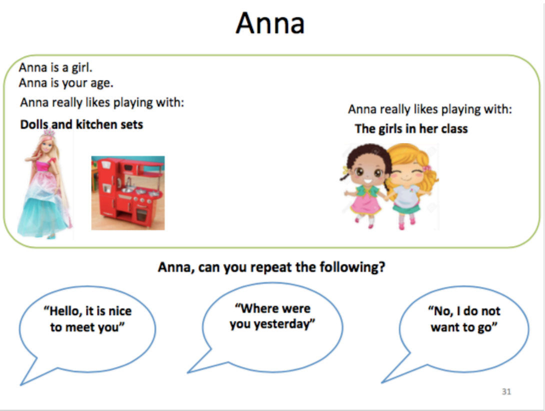

Gender-neutral boy and girl characters

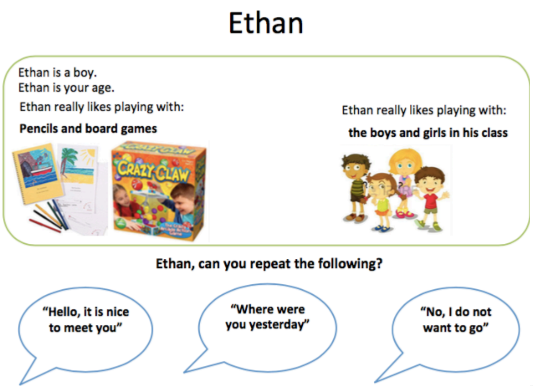

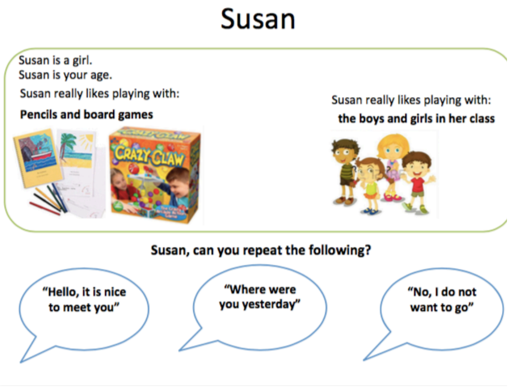

Stereotypically masculine boy and girl characters

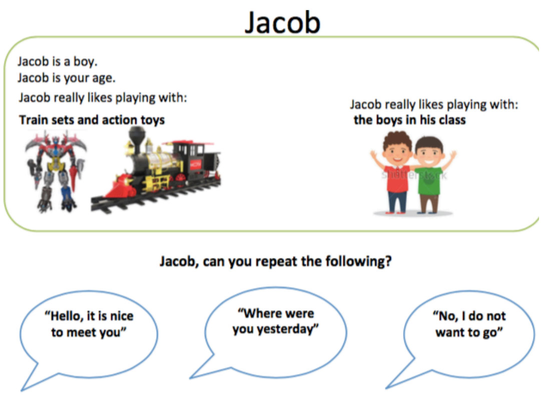

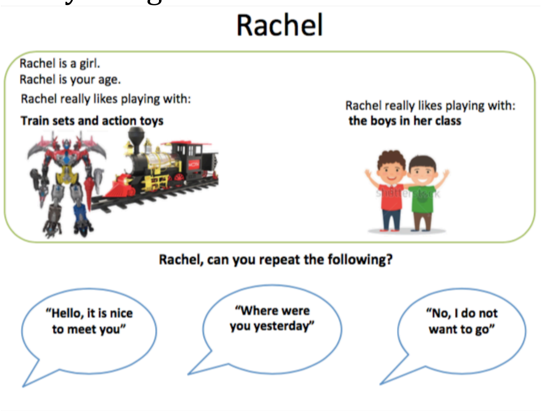

Supplement: Supplementary Material - Stimuli [file rsos190656supp1.pdf]
