# Supplementary figures and images for: Children can control the expression of masculinity and femininity through the voice
Source: R Soc Open Sci. 2019 Jul 17;6(7):190656. doi: 10.1098/rsos.190656 (PMC6689575; doi:10.1098/rsos.190656)

# Fundamental Frequency (F0) Scatterplot of Residuals vs Predicted Values

## Girls

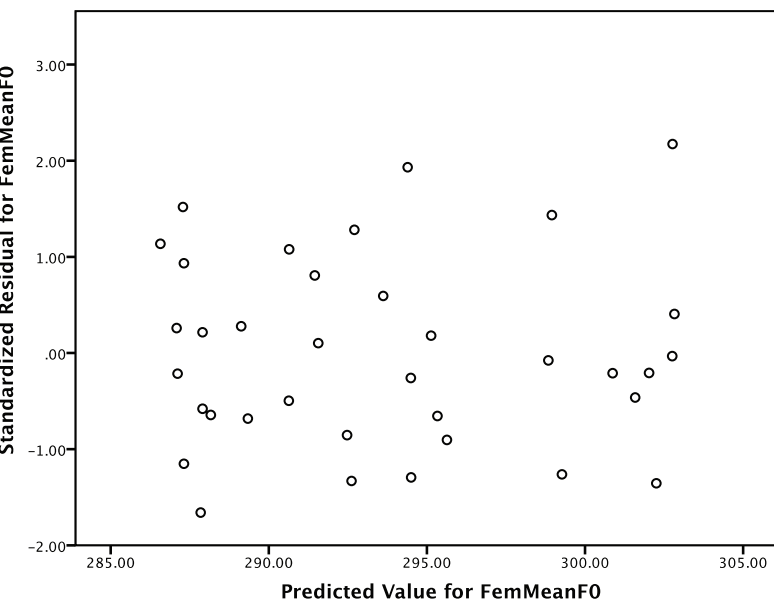

## Boys

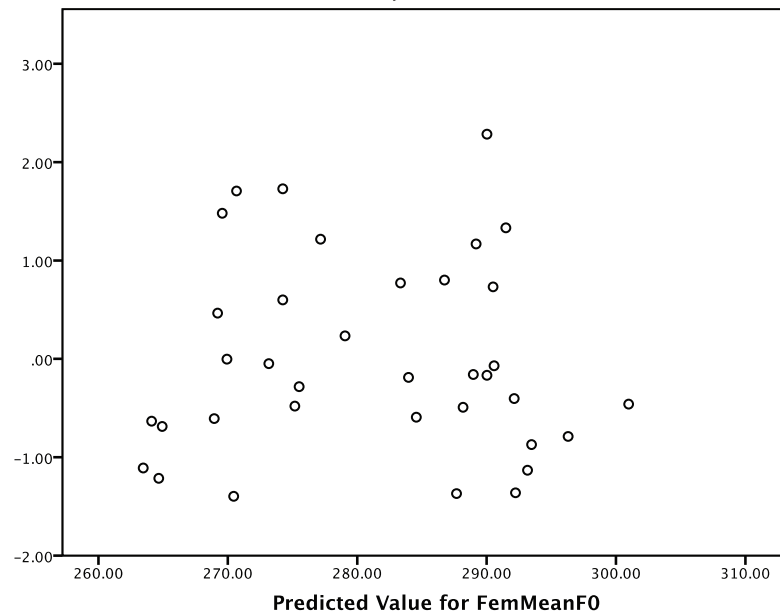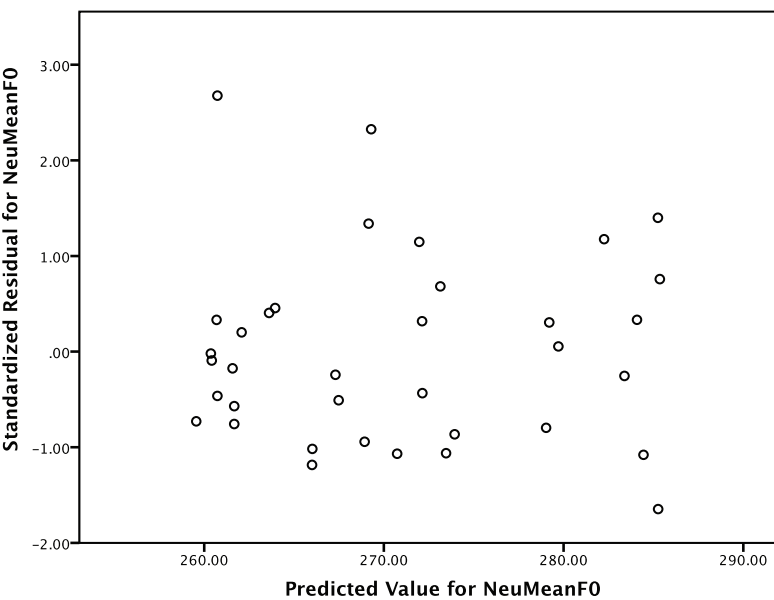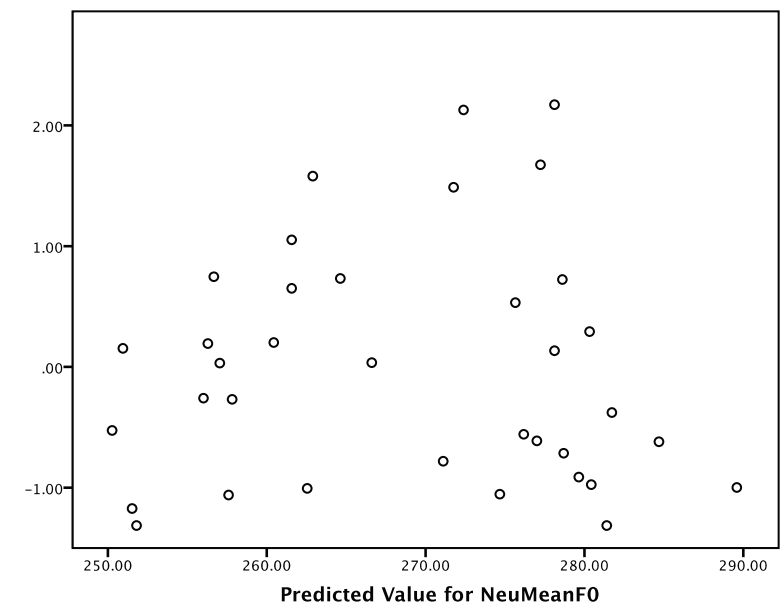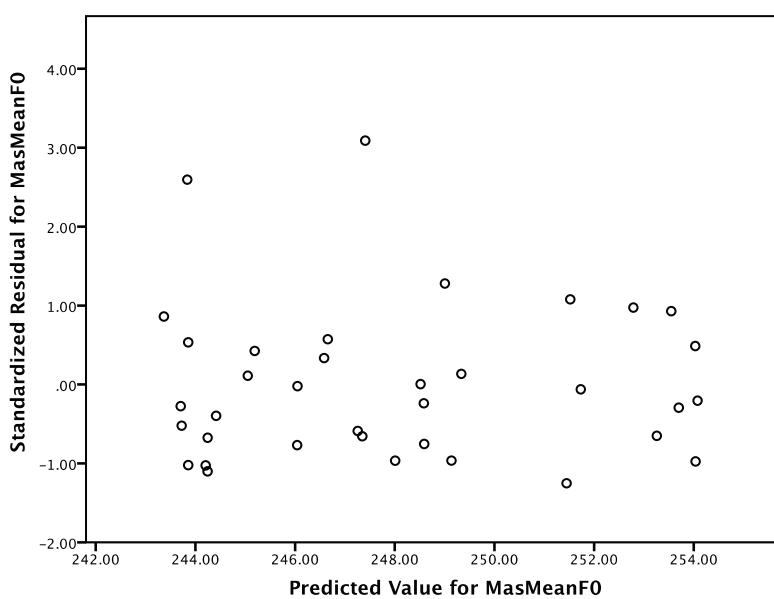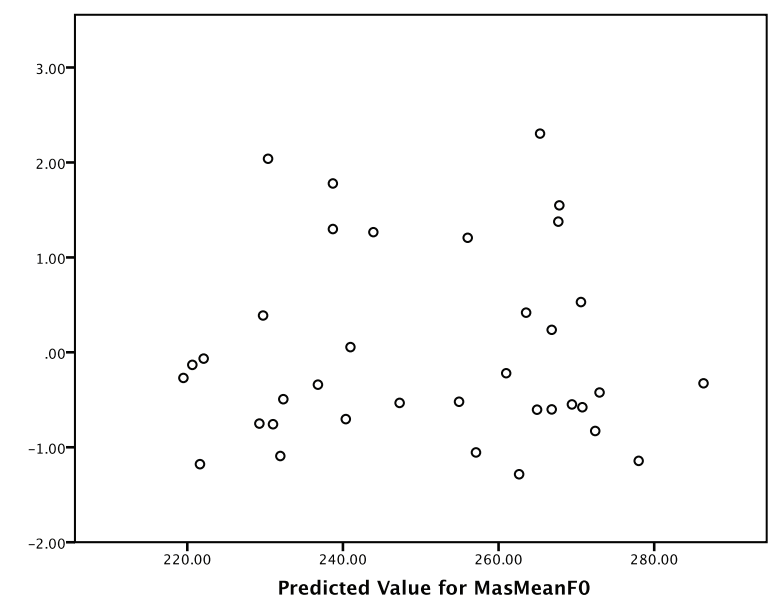

Supplement: Supplementary material - ANCOVA F0 scatterplots Figure S1 [file rsos190656supp2.pdf]

# Formant Spacing ( $\Delta F$ ) Scatterplot of Residuals vs Predicted Values

## Girls

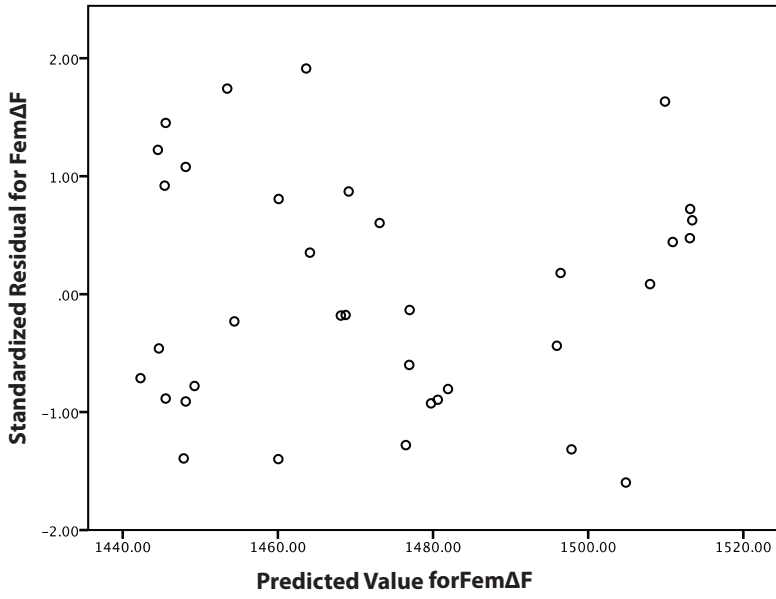

## Boys

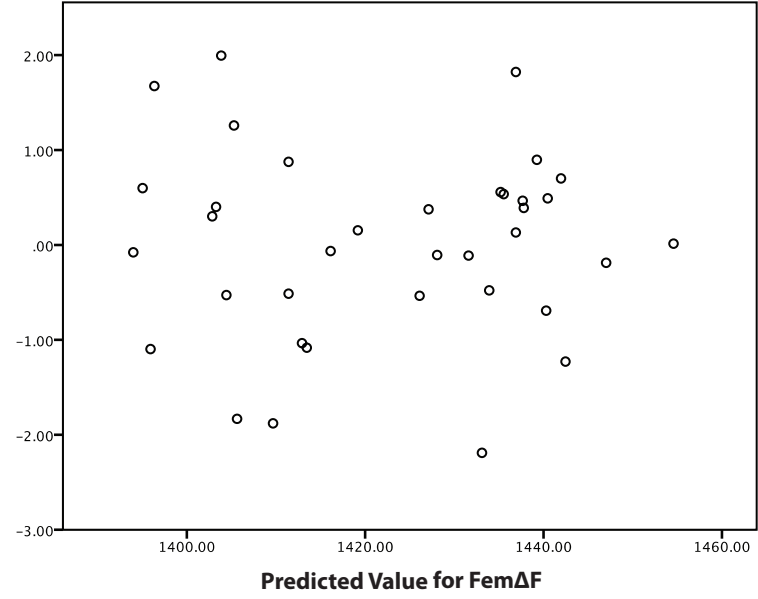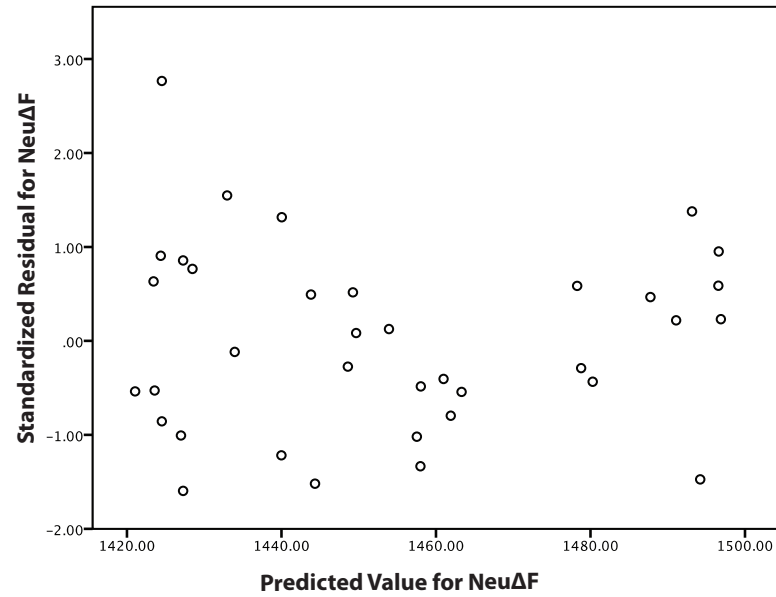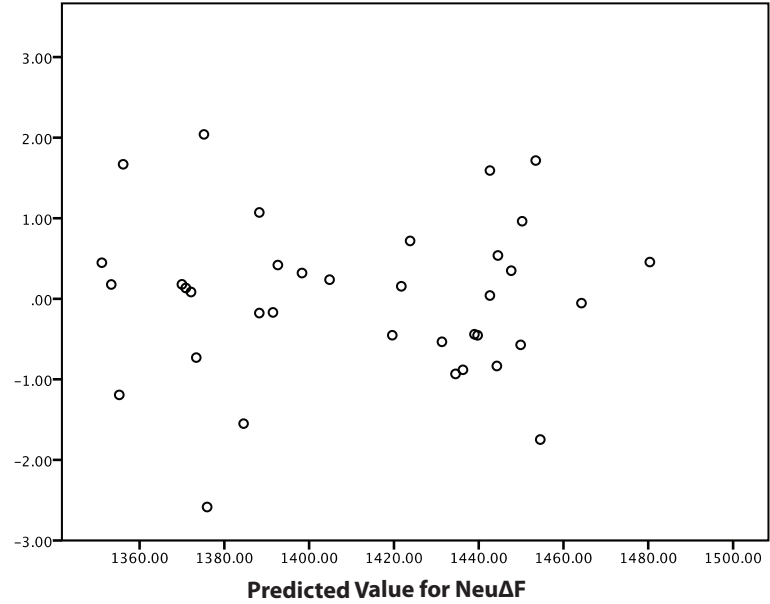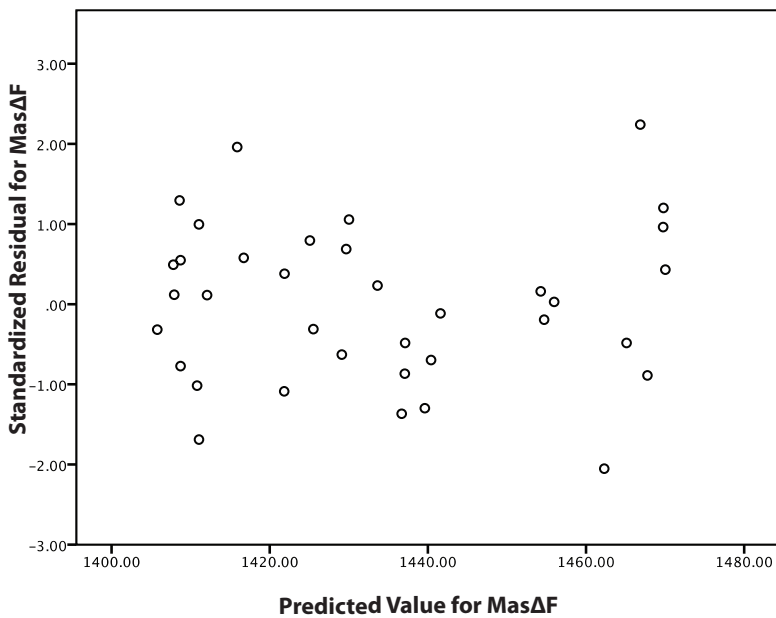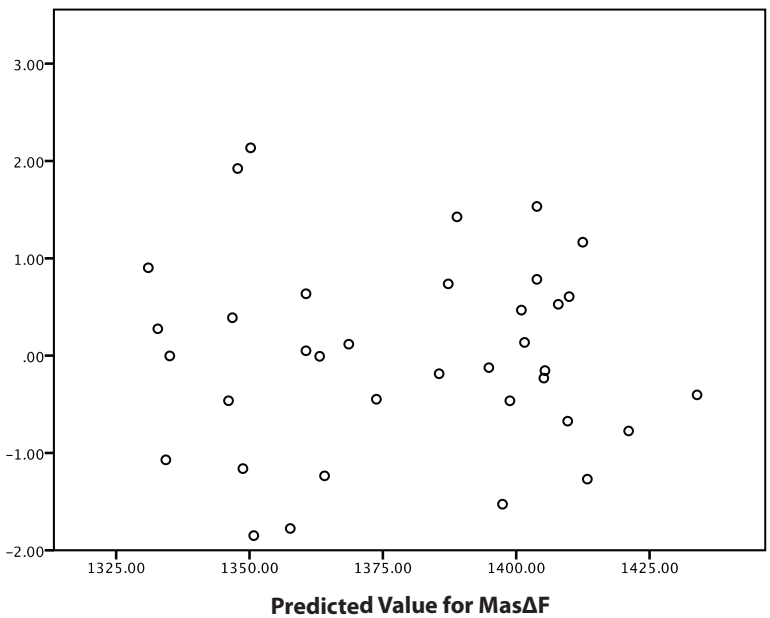

Supplement: Supplementary material - ANCOVA DF scatterplots Figure S2 [file rsos190656supp3.pdf]

Violin Plots of Fundamental Frequency (F0) and Formant Spacing by Character Type

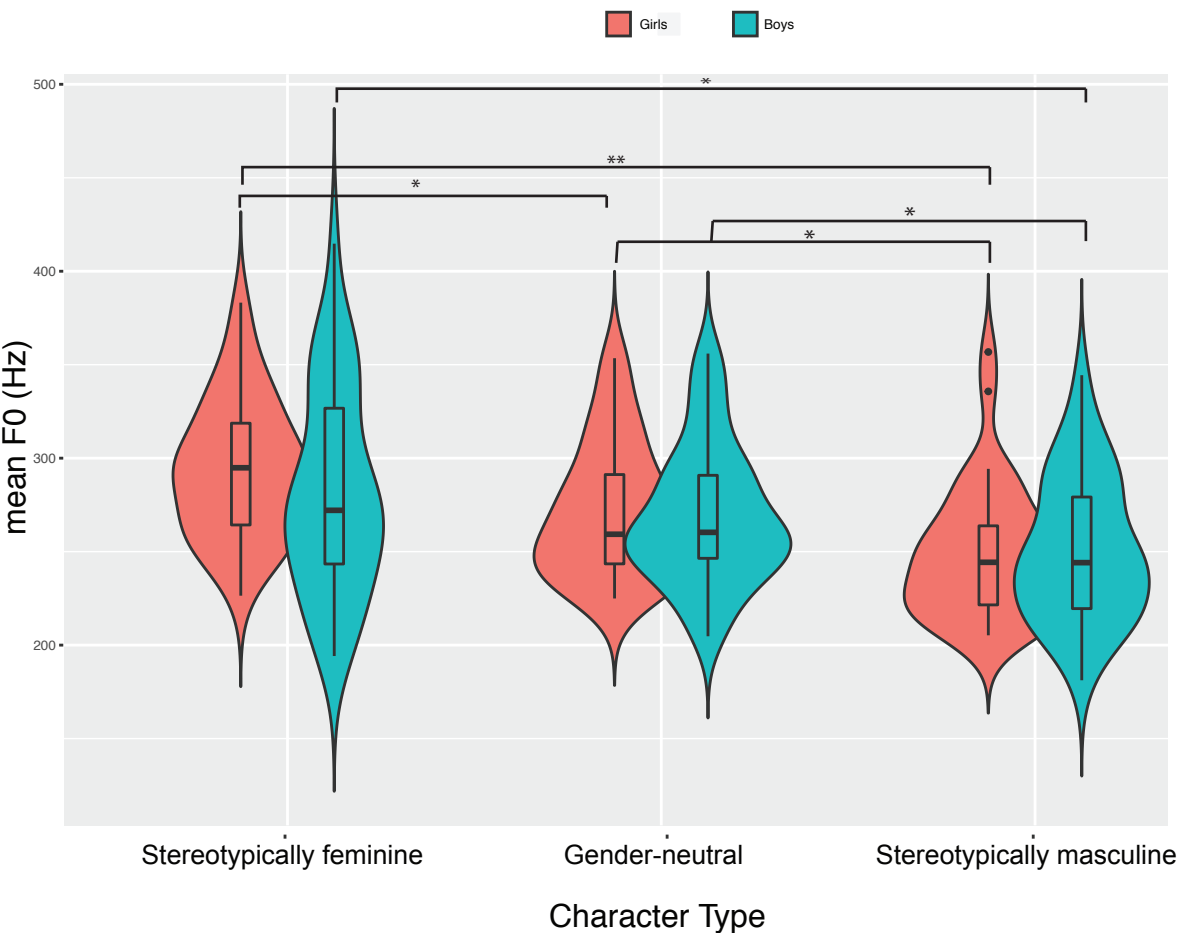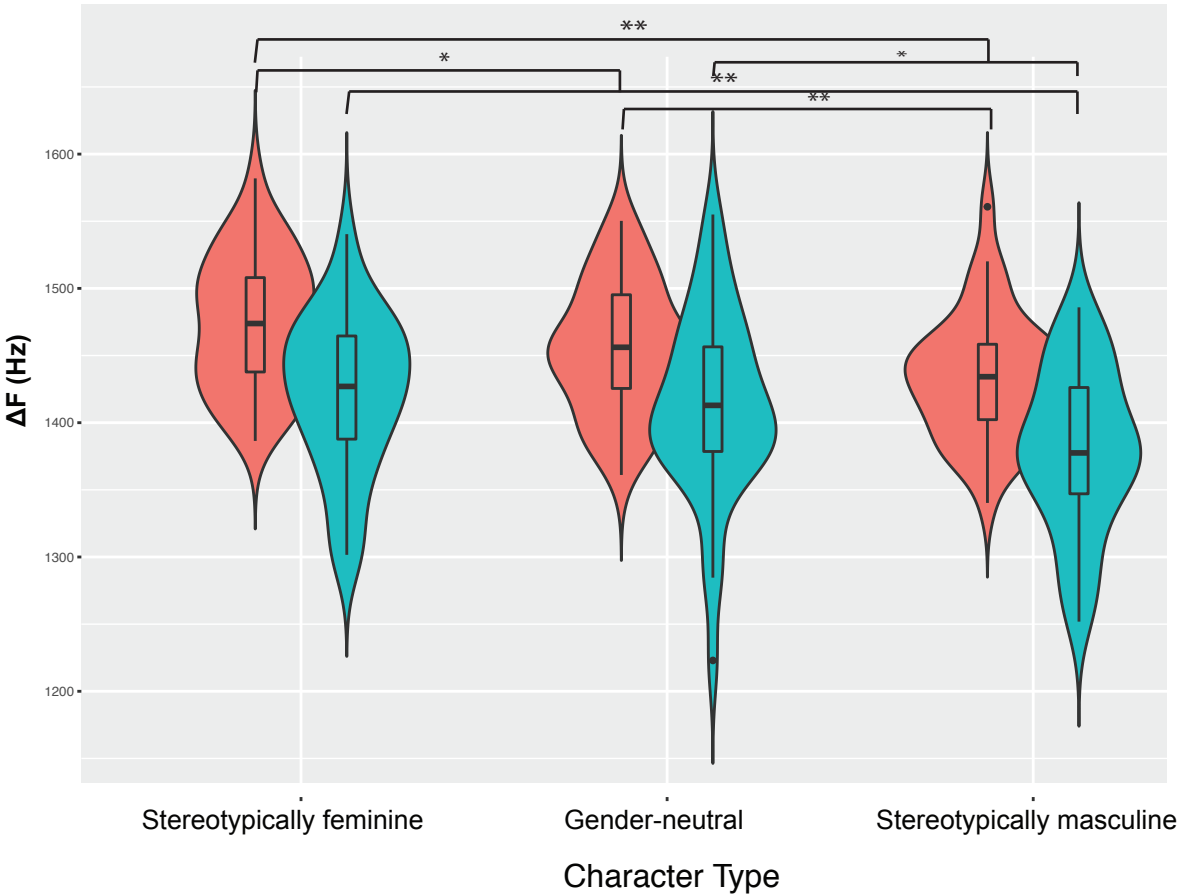

Supplement: Supplementary material - Figure S3 Violin Plots [file rsos190656supp4.pdf]
