# Supplementary material for: Children can control the expression of masculinity and femininity through the voice
Source: R Soc Open Sci. 2019 Jul 17;6(7):190656. doi: 10.1098/rsos.190656 (PMC6689575; doi:10.1098/rsos.190656)
Supplement: Supplementary material - Table S1 Descriptives [file rsos190656supp5.pdf]

Descriptive statistics for the acoustic parameters

We tested n=72 children in total by year group, 24 in UK Years 1&2 (10 girls, mean age=6.48; SE=.14; 14 boys, mean age = 7.0 SE =.11), 22 in Years 3& 4 (12 girls, mean age= 8.6 SE=.13; 10 boys, mean age = 8.4 SE =.17), and 26 in Years 5&6 (14 girls, mean age= 9.97 SE=.14; 12 boys, mean age =9.7 SE =.12). F0 and DF values (Mean, N, SD, Min, Max) for each character type by sex and year group are reported below.

Table S1. Children's F0 and DF values (Mean, N, SD, Min, Max) for each character type (feminine, neutral and masculine) by sex and UK year group.

		Feminine			Neutral			Masculine		
Girls										
		F0	*ΔF*	*aVTL*	*F0*	*ΔF*	*aVTL*	*F0*	*ΔF*	*aVTL*
	Mean	302.7	1509.9	11.6	283.7	1498.6	11.7	253.1	1469.5	11.9
	N	10	10	10	10	10	10	10	10	10
1-2 Yr Group	SD	40.5	47.3	0.4	34.3	35.5	0.3	29.6	52.1	0.4
	Min	252.1	1434.4	11.1	228.2	1433.1	11.3	207.1	1376.3	11.2
	Max	383.2	1581.9	12.2	333.7	1550.3	12.2	289.7	1560.8	12.7
	Mean	294.6	1462.6	12.0	270.1	1432.3	12.2	251.5	1419.9	12.3
	N	12	12	12	12	12	12	12	12	12
3-4 Yr Group	SD	36.1	40.9	0.3	40.3	29.4	0.3	41.1	33.5	0.3
	Min	243.4	1398.4	11.3	224.9	1381.4	11.9	213.9	1376.3	11.9
	Max	365.9	1548.0	12.5	349.9	1470.7	12.7	356.8	1474.3	12.7
	Mean	286.3	1455.9	12.0	262.1	1440.6	12.2	241.4	1419.7	12.3
	N	14	14	14	14	14	14	14	14	14
5-6 Yr Group	SD	36.1	47.2	0.4	30.8	50.2	0.4	34.7	40.9	0.4
	Min	226.4	1386.4	11.4	230.8	1361.1	11.4	205.3	1340.3	11.7
	Max	343.5	1530.3	12.6	353.5	1539.2	12.9	335.7	1498.0	13.1
	Mean	293.6	1473.1	11.9	270.7	1453.9	12.1	248.0	1433.6	12.2
	N	36	36	36	36	36	36	36	36	36
Total	SD	36.9	49.7	0.4	35.3	48.3	0.4	35.1	46.7	0.4
	Min	226.4	1386.4	11.1	224.9	1361.1	11.3	205.3	1340.3	11.2
	Max	383.2	1581.9	12.6	353.5	1550.3	12.9	356.8	1560.8	13.1
Boys										
Year		*F0*	*ΔF*	*aVTL*	*F0*	*ΔF*	*aVTL*	*F0*	*ΔF*	*aVTL*
	Mean	290.4	1453.9	12.1	274.8	1452.1	12.1	273.6	1413.9	12.4
	N	14	14	14	14	14	14	14	14	14
1-2 Yr Group	SD	55.6	41.4	0.3	36.0	60.3	0.5	33.0	40.4	0.4
	Min	217.9	1372.7	11.4	234.3	1351.1	11.3	238.8	1345.4	11.8
	Max	414.7	1540.4	12.8	356.0	1555.0	13.0	344.4	1485.9	13.0
	Mean	290.8	1392.9	12.6	285.3	1408.2	12.4	250.3	1372.0	12.8
3-4 Yr Group	N	10	10	10	10	10	10	10	10	10
	SD	49.6	43.4	0.4	40.8	27.8	0.2	32.9	48.6	0.5

	Min	212.9	1308.7	12.1	226.5	1377.8	11.9	216.2	1298.0	12.0
	Max	368.6	1448.5	13.4	348.7	1466.3	12.7	299.7	1465.2	13.5
	Mean	262.0	1410.3	12.5	247.8	1367.6	12.8	224.8	1344.9	13.1
	N	12	12	12	12	12	12	12	12	12
5-6 Yr Group	SD	57.6	71.8	0.6	26.7	77.7	0.7	35.1	69.9	0.7
	Min	194.2	1301.6	11.5	204.7	1223.0	11.7	181.2	1251.9	12.0
	Max	363.9	1517.1	13.5	284.9	1496.0	14.3	300.4	1464.4	14.0
	Mean	281.1	1422.4	12.3	268.7	1411.7	12.4	250.8	1379.3	12.7
	N	36	36	36	36	36	36	36	36	36
Total	SD	54.9	58.6	0.5	37.1	69.1	0.6	38.9	60.4	0.6
	Min	194.2	1301.6	11.4	204.7	1223.0	11.3	181.2	1251.9	11.8
	Max	414.7	1540.4	13.5	356.0	1555.0	14.3	344.4	1485.9	14.0
