# Supplementary material for: Children can control the expression of masculinity and femininity through the voice
Source: R Soc Open Sci. 2019 Jul 17;6(7):190656. doi: 10.1098/rsos.190656 (PMC6689575; doi:10.1098/rsos.190656)
Supplement: Appendix 1 [file rsos190656supp6.pdf]

Appendix S1 – Formant spacing calculation

Formant spacing (ΔF) was calculated from F1–F4 using the procedure specified by Reby and McComb (2003). When the vocal tract is modelled as a straight uniform tube that is closed at one end and open at the other, the spacing between any two successive formants (ΔF) can be approximated as a constant, and formant frequencies can be plotted as:

(1)
$$F_i = \frac{2(i-1)c}{4aVTL}$$

Where *i* is the formant number, *c* is the speed of sound in a mammal vocal tract (350m/s), *aVTL* is the apparent vocal tract length and *F*i is the frequency of *i*th formant. From (1) and, it follows that:

(2) $$\Delta F = F_{i+1} - F_i = \frac{c}{2aVTL}$$

By replacing 2c/aVTL with ΔF in equation (1), individual formant frequencies can be related to formant spacing Δ*F* by the equation:

(3)
$$F_i = \frac{(2i-1)}{2}\Delta F$$

For each utterance, we can therefore estimate Δ*F*, the overall spacing of the formants, by seeking the best fit for equation (3) to the centre frequency of the first four formants.

The above formula is a good estimate of ΔF because, while individual formants are affected by the shape as well as the length of the vocal tract required to express the different sounds, the formant spacing is an average of adjacent formant differences, and thus provides an overall estimate of spectral dispersion which is less sensitive to such deviations.

Additionally, as ΔF is determined by, and inversely correlated to, the length of the vocal tract of the speaker (Titze, 1994), it follows from (2) that the apparent vocal tract length can be estimated as aVTL = c/2(ΔF). Apparent Vocal Tract Length (aVTL) was calculated because it can be expressed in cm and therefore gives a better illustration of the scale of the manipulations performed than ΔF (which is expressed in Hz).

While the quarter-wave resonator is an accurate model for the unconstricted schwa sound, the production of other vowels involve constrictions in the oral tract, affecting individual formant values and therefore requiring more complex models. It is worth noting, however, that subject differences in vocal tract length will still be reflected in ΔF differences for these vowels, and that averaging formants in connected speech leads to central vowel values, that reflect physical speaking (as opposed to resting) vocal tract length quite accurately (Titze, 1994).
